# Route- and sex-dependent toxicity of clinical-stage silver nanoparticles in mice

**DOI:** 10.1039/d6nr00957c

**Published:** 2026-09-07

**Authors:** Buerlan Yeerkenbieke, Osmane Camara, Ruxandra-Ioana Cipu, Miryam Warwar, Laura Zediu, Rui Zhang, Eva Miriam Buhl, Stephan Rütten, Bea Becker, Jens Köhler, Joachim Mayer, Fabian Kiessling, Twan Lammers, Bo Han, Ba Xuan Hoang, Roger M. Pallares

**Affiliations:** a Institute for Experimental Molecular Imaging, RWTH Aachen University Hospital Aachen 52074 Germany rmoltopallar@ukaachen.de; b Ernst Ruska-Centre for Microscopy and Spectroscopy with Electrons, Forschungszentrum Jülich GmbH Jülich 52425 Germany; c Faculty of Medical Engineering, National University of Science and Technology Politechnica Bucharest Bucharest 011061 Romania; d Electron Microscope Facility, Institute for Pathology, RWTH Aachen University Hospital Aachen 52074 Germany; e DWI – Leibniz Institute for Interactive Materials Aachen 52074 Germany; f Department of Surgery, Keck School of Medicine, University of Southern California Los Angeles California 90033 USA baxuanho@usc.edu

## Abstract

Silver nanoparticles (AgNPs) are attractive therapeutic agents for highly specific applications; however, safety concerns about organ-specific toxicity following systemic exposure limit their development. Here, we present a multi-endpoint preclinical safety assessment of the clinical-stage silver nanomedicine Nowarta110 following repeated dermal topical, oral mucosal, and intravaginal administration in mice. The safety profile of Nowarta110 was evaluated through both longitudinal in-life monitoring and comprehensive terminal analyses, including hematology, serum biochemistry, urinalysis, organ weight measurements, detailed histopathological examination, and toxicokinetic profiling. Across all endpoints, no treatment-related toxicological effect was observed. Clinical pathology parameters, organ weights, and histopathological findings were consistent with controls, with no sex-dependent differences identified. Notably, plasma silver concentrations remained below the mass spectrometry limit of quantification at all time points, indicating no detectable systemic exposure. This lack of circulating silver supports the favorable safety profile of Nowarta110 across multiple local administration routes. Collectively, these findings further support the safety profile of this clinically-stage silver nanomedicine for local therapeutic applications.

## Abstract 2

We present a multi-endpoint preclinical safety assessment of the clinical stage silver nanomedicine Nowarta110 following repeated dermal topical, oral mucosal, and intravaginal administration in mice.

## Graphical abstract

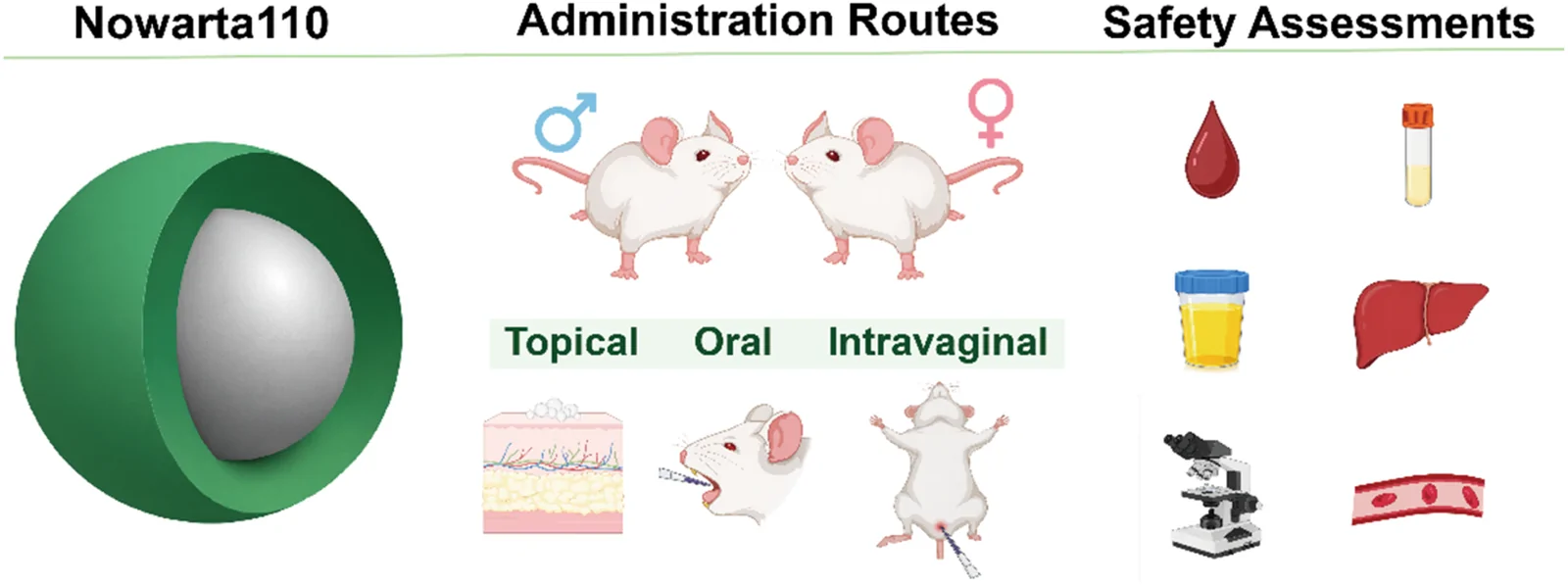

## 1. Introduction

Silver has long been recognized as a naturally occurring element with notable antimicrobial activity, and its medical use has been documented throughout history.^1–3^ The antimicrobial effects of ionic and metallic silver involve several mechanisms, including free radical generation, DNA damage, and disruption of cellular membranes.^4,5^ Silver nanoparticles (AgNPs) are metallic silver particles with diameters ranging from 1 to 100(0) nm, and are among the most successful silver formulations, being used in a wide range of applications from healthcare to textiles.^6,7^ Their nanoscale dimensions give rise to a high surface-to-volume ratio, which enhances chemical reactivity and facilitates interactions with biological environments. These features contribute to the well-known antimicrobial and wound-healing properties of AgNPs.^8,9^ Consistent with these properties, wound dressings based on AgNPs have been widely developed and commercialized, reflecting the continued clinical relevance of silver-derived materials.^10,11^ Beyond antimicrobial applications, AgNPs exhibit characteristic plasmonic and photothermal behaviors that originate from localized surface plasmon resonances, an intrinsic physicochemical property of metallic silver at the nanoscale.^12,13^ These optical features have motivated research into AgNPs as preclinical agents for photoacoustic imaging and photothermal therapy,^14–16^ alongside early clinical investigations of their use in the photothermal treatment of skin conditions.^17^

Currently, more than a dozen silver-based nanoformulations are being investigated in clinical trials.^17^ Due to concerns regarding their long-term accumulation in highly perfused and fenestrated organs, the clinical development of AgNPs has largely focused on topical administration. Among these, Nowarta110 is one of the most advanced and clinically relevant formulations. It consists of a silver nanocore surrounded by a fig-extract shell, enabling synergistic antiviral activity from both the fig extract and the AgNPs, as well as enhanced dermal penetration attributable to its nanoscale size and physicochemical properties.^18,19^ As a result, Nowarta110 was successfully evaluated in a phase I/II clinical study for the treatment of viral-induced plantar warts, and a phase III clinical trial is currently planned.^18^ In addition, Nowarta110 has demonstrated therapeutic efficacy in preclinical models of papillomavirus-associated cervical cancer and melanoma.^20,21^

The renewed interest in AgNPs as therapeutic agents underscores the need for a comprehensive evaluation of their safety and long-term biological behavior.^22–25^ However, most preclinical safety studies continue to prioritize intravenous administrations, despite this route being rarely used for AgNPs, while comparatively overlooking more prevalent routes, such as topical or oral administration. Moreover, accumulating evidence over the past decade indicates sex-dependent differences in nanomaterial and nanomedicine responses,^26–28^ highlighting the necessity of incorporating sex as a biological variable in AgNP safety assessments.

In this study, prompted by the advanced clinical development of Nowarta110 and its potential for expanded therapeutic applications, we systematically characterized its toxicological profile in murine models. We evaluated route-dependent safety following dermal topical, oral mucosal, and intravaginal administration using comprehensive hematological, serum biochemical, and urinalysis examinations, alongside assessments of food consumption and body weight, and detailed pathological analyses. Our findings indicate that Nowarta110 does not elicit treatment-related toxicity nor sex-dependent differences in biological responses. Importantly, blood analyses confirmed that three administration routes do not lead to systemic exposure to the nanoformulation. Collectively, these results further support the continued investigation of Nowarta110 as a promising silver-based nanomedicine for dermal topical, oral mucosal, or intravaginal use.

## 2. Experimental methods

### 2.1. Formulation and characterization

Nowarta110 was obtained from Nowarta Biopharma (CA, USA). The morphology of the nanoformulation was examined by transmission electron microscopy (TEM) using a Hitachi microscope operated at 100 kV. For TEM analysis, AgNP suspensions were drop-cast onto carbon-coated copper grids and left to dry under ambient conditions before imaging. High resolution TEM imaging was performed using an aberration-corrected FEI Titan 80–300 transmission electron microscope (TITAN-T) operated at 300 kV. Particle size distribution was determined based on TEM micrographs. Elemental composition was analyzed by scanning transmission electron microscopy coupled with energy-dispersive X-ray spectroscopy (STEM-EDS). Measurements were carried out using a Quattro S scanning electron microscope (Thermo Fisher Scientific, USA) equipped with a detector, operated at an accelerating voltage of 10 kV under high-vacuum conditions. Surface chemical features of the AgNPs were characterized by Fourier-transform infrared (FTIR) spectroscopy using a Spectrum 3 FTIR spectrometer (PerkinElmer, USA). Before analysis, samples were lyophilized with an Alpha 2–4 LD PLUS freeze dryer (Martin Christ, Germany), and spectra were collected in the range of 4000–400 cm^−1^.

### 2.2. Animals

Specific pathogen-free BALB/c mice were used in this study. Five-week-old animals were purchased from Envigo and acclimated for 7 days before study initiation. At the start of dosing, mice were 7–8 weeks of age and weighed approximately 18–20 g. Animals were housed in wire cages with up to 5 mice per cage in a temperature-controlled room (65–76 °F) with a relative humidity of 50% ± 20% and a 12-hour light–dark cycle. Standard rodent chow and water were provided *ad libitum* throughout the acclimation and experimental periods. Each animal was assigned a unique identification number using ear tags.

All animal procedures were conducted at BATTS Laboratories (USA) and were reviewed and approved by the BATTS Laboratories Institutional Animal Care and Use Committee (IACUC). Animal care and experimental procedures were conducted in accordance with the Guide for the Care and Use of Laboratory Animals. The study was also conducted in accordance with the harmonized OPPTS test guideline, derived in part from 40 CFR 798.2650 for toxicity assessment.

### 2.3. Study design and dosing regimen

The study was conducted as a repeated-dose toxicity assessment to evaluate the potential toxicological effects of Nowarta110 following different routes of administration. Mice were randomly assigned to four experimental groups, including one control group and three treatment groups receiving the nanomedicine *via* dermal topical, oral mucosal, or intravaginal administration. The intravaginal administration group included female mice (*n* = 10), while the oral and topical administration groups included male and female mice (*n* = 10 per sex). A single control group (*n* = 10 per sex) was used for comparison across all treatment groups. Nowarta110 was administered once daily for 12 consecutive weeks. For intravaginal administration, female mice received a vaginal dose of 7–10 µL per mouse per day. For oral mucosal administration, a dose of 40 µL per mouse per day was applied to the oral cavity. For dermal topical administration, 340 µL of formulation was applied daily to a defined area of skin (approximately 2 cm in diameter). Dose levels were determined based on the results of a previously conducted dose-finding non-Good Laboratory Practice (non-GLP) efficacy study, which led to the dose used in the clinical evaluation trial of Nowarta110.^18^ Moreover, comparable doses had been administered *via* the same routes in earlier studies exploring other inorganic nanoparticles.^29–31^ All animals were observed daily for general clinical signs throughout the study. Body weight and food consumption were monitored on a weekly basis during the study period.

### 2.4. Hematological, serum biochemical, and urinalysis examinations

The different analyses were performed on all animals, including controls, at baseline, week 5, and at the end of the study. Animals were fasted overnight prior to blood collection. Hematological evaluation was performed to assess general hematopoietic status and potential treatment-related alterations in blood cell populations, including measurements of white blood cell count, lymphocytes, monocytes, granulocytes, lymphocyte percentage, monocyte percentage, red blood cell count, hemoglobin, hematocrit, mean corpuscular volume, mean corpuscular hemoglobin, mean corpuscular hemoglobin concentration, platelet count, and mean platelet volume.

Biochemistry assessment encompassed markers of liver and renal function, electrolyte balance, and metabolic status, including alanine aminotransferase, albumin, alkaline phosphatase, amylase, calcium, phosphorus, creatinine, globulin, glucose, sodium, total bilirubin, total protein, blood urea nitrogen, and potassium.

Urinalysis was conducted to assess renal function and metabolic alterations, including measurements of pH, protein, glucose, ketone bodies, blood, urobilinogen, bilirubin, nitrite, and leukocytes.

### 2.5. Necropsy and histopathological examination

At necropsy, organ weights were recorded for all animals, including the eyes, brain, mesenteric lymph nodes, lungs, heart, liver, pancreas, spleen, stomach, small intestine, large intestine, kidneys, and urinary bladder. In addition, testes were weighed in male mice, and ovaries and mammary glands were weighed in female mice. For histopathological examination, the following organs and tissues, or representative samples thereof, were collected and preserved in 10% buffered formalin immediately after necropsy: the digestive system (stomach, small intestine, large intestine, liver, and pancreas), the nervous system (brain and eyes), the respiratory system (lungs), the cardiovascular and hemopoietic systems (heart, lymph nodes, and spleen), and the urogenital system (kidneys, urinary bladder, testes, uterus, ovaries, mammary glands, and external genitalia). In addition, all gross lesions and masses, as well as skin samples, were collected for histopathological evaluation. All tissues designated for microscopic examination were fixed for no less than 48 hours before trimming. The histopathological assessment was conducted by veterinarians in a certified *in vivo* laboratory.

### 2.6. Toxicokinetic evaluation

A separate group of mice was used for the toxicokinetic study. The dose levels of Nowarta110 used in this study were identical to those employed in the primary intravaginal, oral, and topical toxicity studies. For the intravaginal toxicokinetic study, 18 female mice were used. Blood samples were collected from two animals at each time point, including 0, 1, 3, 6, 9, 12, and 24 hours, as well as at 5 and 12 weeks following dosing. For the oral and dermal topical toxicokinetic studies, blood samples were collected from three male and three female mice at each time point.

Plasma silver concentrations were determined using a validated inductively coupled plasma mass spectrometry (ICP-MS) method, with total silver used as a surrogate marker of systemic exposure to Nowarta110. The limit of detection and limit of quantification of the method were 6.8 ng mL^−1^ and 22.6 ng mL^−1^, respectively. Mass spectrometry is widely used in the quantification of heavy elements in biological and complex samples.^32–37^

### 2.7. Statistical analysis

All data analysis and visualization were performed using R. For hematology and serum biochemistry heatmaps, each group and time point was compared with the pooled week 0 baseline using linear models. *P* values were adjusted using the Benjamini–Hochberg false discovery rate method. Adjusted *p* values < 0.01 were considered statistically significant. Significant comparisons were additionally required to meet an effect size threshold of |log10 fold change| ≥ 0.15. Organ weights were analyzed using the Welch *t*-test. *P* values were adjusted for multiple comparisons using the Holm method, and adjusted *p* values < 0.05 were considered statistically significant.

## 3. Results

### 3.1. Physicochemical characterization of Nowarta110

**Fig. 1:**
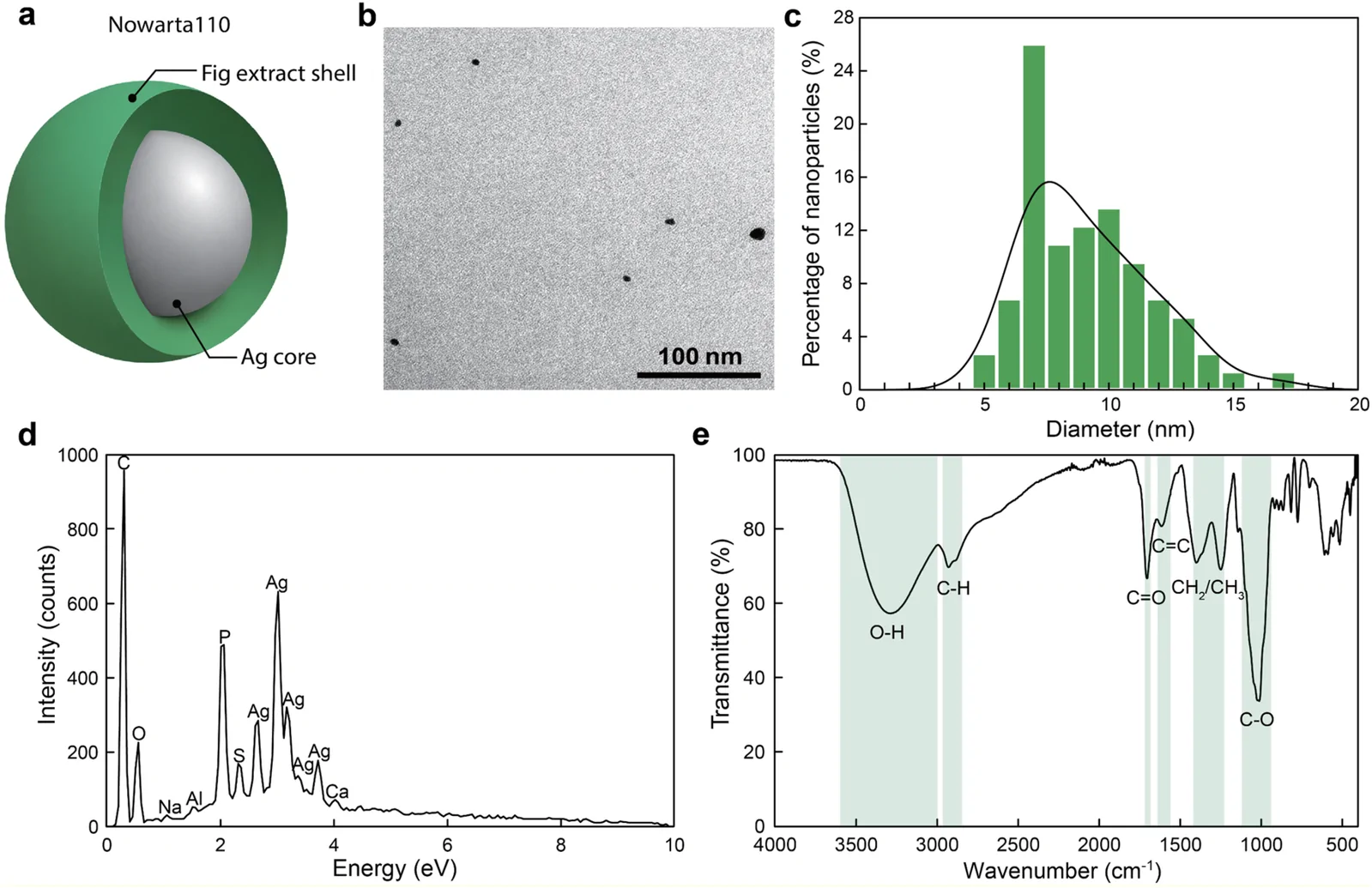
Physicochemical characterization of Nowarta110. (a) Schematic representation, (b) representative TEM micrograph, (c) particle size distribution, (d) EDS spectrum, and (e) FTIR spectrum of Nowarta110. Abbreviations: O–H (hydroxyl stretching vibration), C–H (aliphatic C–H stretching vibration), C=O (carbonyl stretching vibration), C=C (alkene vibration), CH_2_/CH_3_ (CH_2_/CH_3_ bending), and C–O (C–O stretching vibration).

Nowarta110 was obtained from Nowarta Biopharma (CA, USA) and used as received. The nanomedicine consists of a AgNP core coated with an organic layer derived from fig extract, forming a core–shell structure (Fig. 1a). Transmission electron microscopy revealed predominantly spherical, well-dispersed nanoparticles in the nanometer size range (Fig. 1b) with an average diameter of 7 ± 10 nm (Fig. 1c). High-resolution electron microscopy uncovered the crystal structure of the core, with a fast Fourier transform pattern exhibiting reflections indexed to the (020) and (002) planes of metallic, face-centered cubic silver (Fig. S1).

Elemental analysis further confirmed silver as the principal constituent of the nanoparticle core, as evidenced by characteristic silver (Ag) signals in the EDS spectrum (Fig. 1d). The surface chemistry of Nowarta110 was examined by FTIR spectroscopy, which revealed multiple features consistent with an organic surface layer (Fig. 1e). A broad band at 3700–3000 cm^−1^ was attributed to O–H stretching vibrations,^38^ while signals in the 3000–2800 cm^−1^ range correspond to aliphatic C–H stretching vibration.^39^ The band observed at 1730–1680 cm^−1^ is consistent with a carbonyl (C=O) stretching vibration.^40^ An additional band in the 1650–1600 cm^−1^ region corresponds to C=C stretching vibrations.^39^ Additional features in the 1500–1300 cm^−1^ region may be associated with CH_2_ or CH_3_ bending vibrations and bands within 1175–940 cm^−1^ are characteristic of C–O-related vibrations,^39,41^ including those found in carbohydrates and phenolic structures. Taken together, these observations confirm that Nowarta110 consists of nanoscale silver particles coated with an organic surface layer.

### 3.2. Hematological analysis

The safety profile of Nowarta110 was evaluated through a repeated-dose toxicity assessment (treatment once daily for 12 consecutive weeks) *via* dermal topical, oral mucosal, or intravaginal administrations. These studies were performed in both male and female mice (ten animals per group) at optimal dose levels identified in previous studies with Nowarta110 and other similar inorganic nanoparticles (see the Experimental methods section for further details). Hematological analysis was carried out to assess overall hematopoietic status and the potential effects of the treatments on blood cell populations. Parameters evaluated included total white blood cell counts; differential leukocyte counts expressed as absolute values and percentages (lymphocytes, monocytes, and granulocytes); red cell quantity and oxygen-carrying capacity (red blood cell count, hemoglobin concentration, and hematocrit); red blood cell indices (mean corpuscular volume, mean corpuscular hemoglobin, and mean corpuscular hemoglobin concentration); and platelet parameters (platelet count and mean platelet volume).

**Fig. 2:**
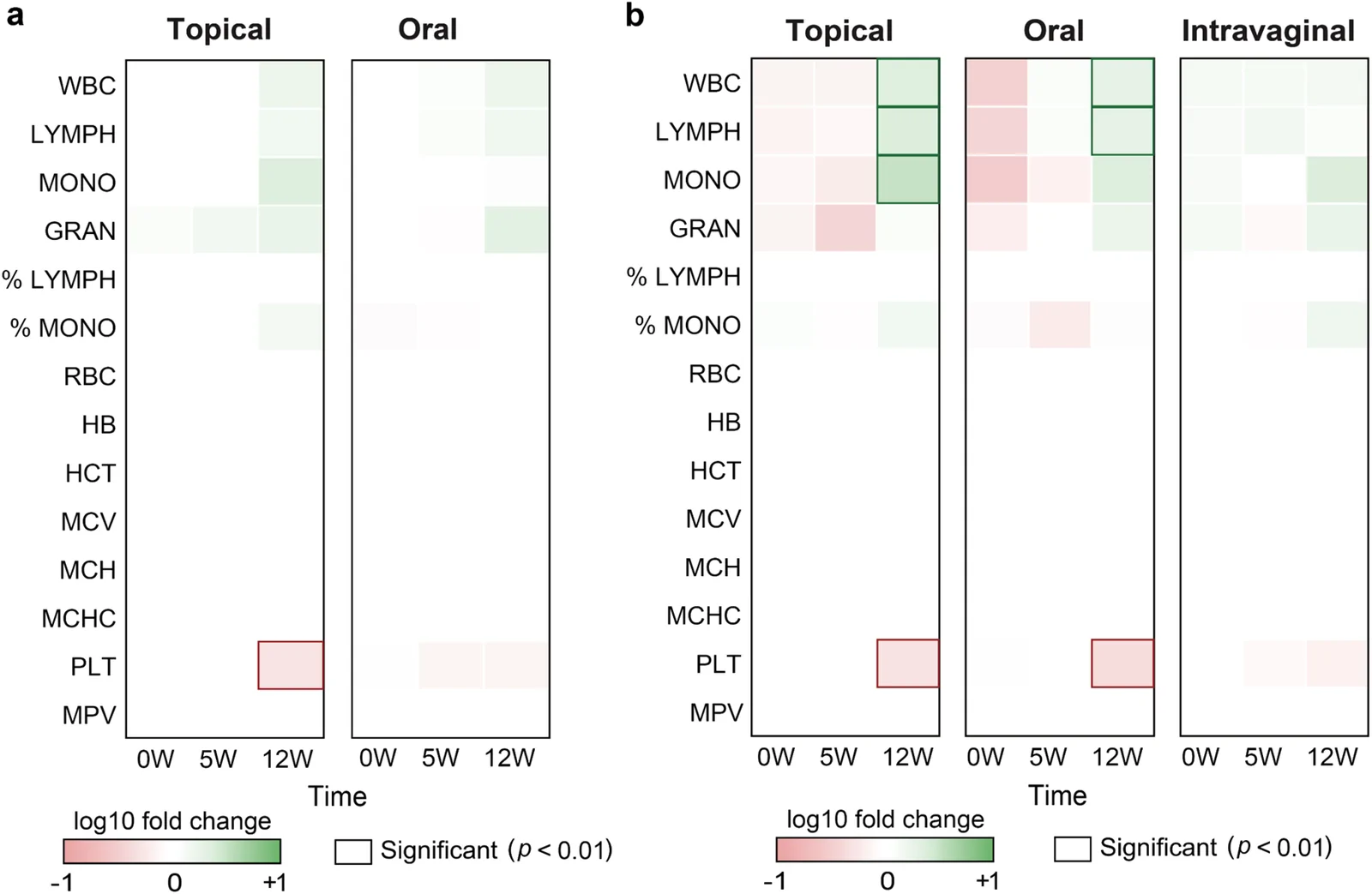
Hematological profiling during Nowarta110 treatments. (a) Male and (b) female hematological heatmaps across administration routes and time points. Color scale indicates log10 fold change compared to relative to the pooled week 0 baseline; boxed cells indicate FDR-adjusted *p* values < 0.01 and |log10 fold change| ≥ 0.15. Abbreviations: white blood cell count (WBC), lymphocytes (LYMPH), monocytes (MONO), granulocytes (GRAN), lymphocyte percentage (% LYMPH), monocyte percentage (% MONO), red blood cell count (RBC), hemoglobin (HB), hematocrit (HCT), mean corpuscular volume (MCV), mean corpuscular hemoglobin (MCH), mean corpuscular hemoglobin concentration (MCHC), platelet count (PLT), and mean platelet volume (MPV).

Notably, hematological parameters remained largely stable across treatment groups and time points (Fig. 2 and Table S1). In male mice, only minor fluctuations were observed, with no consistent pattern suggestive of treatment-related effects. Similarly, in females, although a small number of parameters reached statistical significance at isolated time points, these changes were not sustained and showed no clear association with treatment. For example, increases in white blood cell lymphocyte counts were observed at 12 weeks in females receiving topical and oral administration; however, these changes were not accompanied by consistent alterations across the broader leukocyte profile at the same time point. Overall, the observed hematological variations were inconsistent, lacked dose- or route-dependence, and were comparable to background biological variability, indicating no treatment-specific hematological toxicity.

### 3.3. Biochemical serum profiling

Biochemical analysis of soluble serum constituents was conducted to evaluate potential treatment-induced alterations in organ function, tissue injury, and metabolic stress. The assessed parameters included markers of liver function (alanine aminotransferase, alkaline phosphatase, total bilirubin, albumin, globulin, and total protein); renal function (creatinine and blood urea nitrogen); pancreatic and digestive function (amylase); carbohydrate and energy metabolism (glucose); electrolyte balance (sodium and potassium); and mineral homeostasis (calcium and phosphorus).

**Fig. 3:**
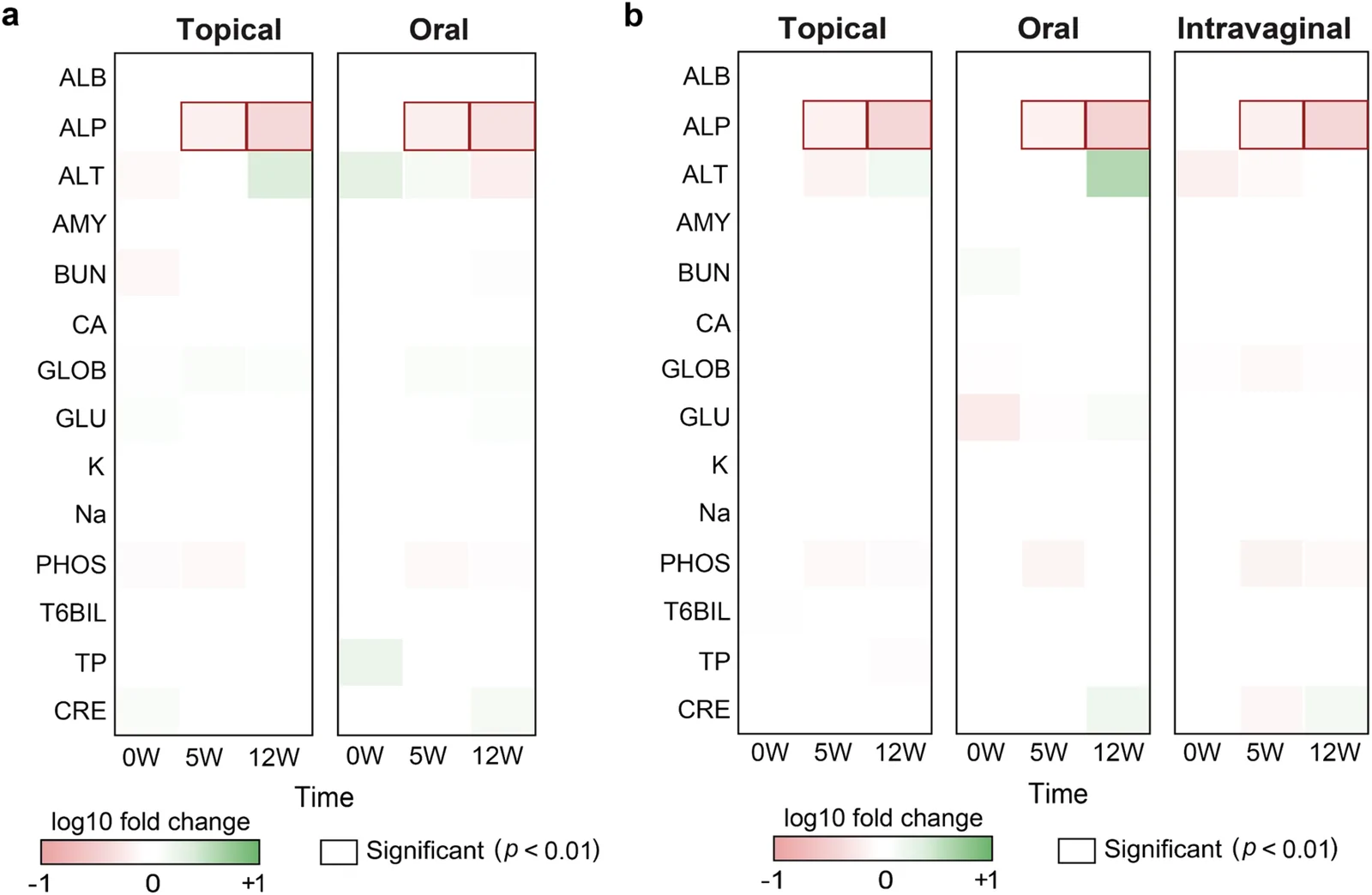
Biochemical analysis during Nowarta110 treatments. (a) Male and (b) female biochemistry heatmaps across administration routes and time points. Color scale indicates log10 fold change compared to relative to the pooled week 0 baseline; boxed cells indicate FDR-adjusted *p* values < 0.01 and |log10 fold change| ≥ 0.15. Abbreviations: alanine aminotransferase (ALT), albumin (ALB), alkaline phosphatase (ALP), amylase (AMY), calcium (Ca), phosphorus (PHOS), creatinine (CRE), globulin (GLOB), glucose (GLU), sodium (Na), total bilirubin (T6BIL), total protein (TP), blood urea nitrogen (BUN), and potassium (K).

Biochemical analysis of serum constituents revealed minimal alterations across treatment groups and time points (Fig. 3 and Table S2). Among the liver function parameters examined, only alkaline phosphatase showed statistically significant changes, and these were modest decreases observed across all administration routes. Serum alkaline phosphatase comprises multiple isoenzymes and its levels may vary under physiological conditions, such as nutritional status or hormonal modulation.^42^ Importantly, no concurrent alterations were detected in other liver-associated biomarkers, including alanine aminotransferase, total bilirubin, albumin, globulin, or total protein. In the absence of concordant changes in complementary hepatic indicators, isolated reductions in serum alkaline phosphatase are not generally considered indicative of hepatic injury.^43^ No coordinated changes were observed in renal markers, electrolyte parameters, or metabolic indicators. Overall, the biochemical findings do not support evidence of systemic toxicity associated with Nowarta110 treatment.

### 3.4. Urinalysis

Urinalysis was subsequently carried out to provide complementary information on renal excretory function, urinary tract integrity, and systemic homeostasis. Even though serum biochemistry analysis characterizes circulating markers of organ function, urinalysis can detect early or subtle renal and metabolic alterations, thereby contributing to a more comprehensive evaluation of treatment-related effects. Specifically, the analysis included evaluation of physical and chemical urine properties (pH); markers of renal and urinary tract integrity (protein, blood, leukocytes); metabolic and endocrine indicators (glucose and ketone bodies); hepatic and hematologic markers (bilirubin and urobilinogen); and infection-related markers (nitrite). The complete urinalysis dataset is provided in Table S3.

The parameters did not exhibit any differences with respect to group, time point, sex, or route of administration. Urine pH was consistently around 5 throughout groups. Markers of renal and urinary tract injury, including protein, blood, and leukocytes, were negative in all animals. Infection-related markers remained consistently negative. Metabolic parameters did not indicate alterations in carbohydrate or energy metabolism. Urine bilirubin and urobilinogen levels were unremarkable, with no trends related to sex or route of administration observed. Altogether, urinalysis did not reveal any renal, urinary tract, or systemic metabolic toxicity associated with repeated exposure to Nowarta110.

### 3.5. Food consumption and body weight

Food consumption and body weight were monitored throughout the 12-week study as general indicators of animal well-being, nutrition status, and systemic tolerability. Variations in food intake can indicate stress or treatment-related adverse effects before the onset of overt clinical signs, whereas changes in body weight are indicative of overall health and metabolic balance.

**Fig. 4:**
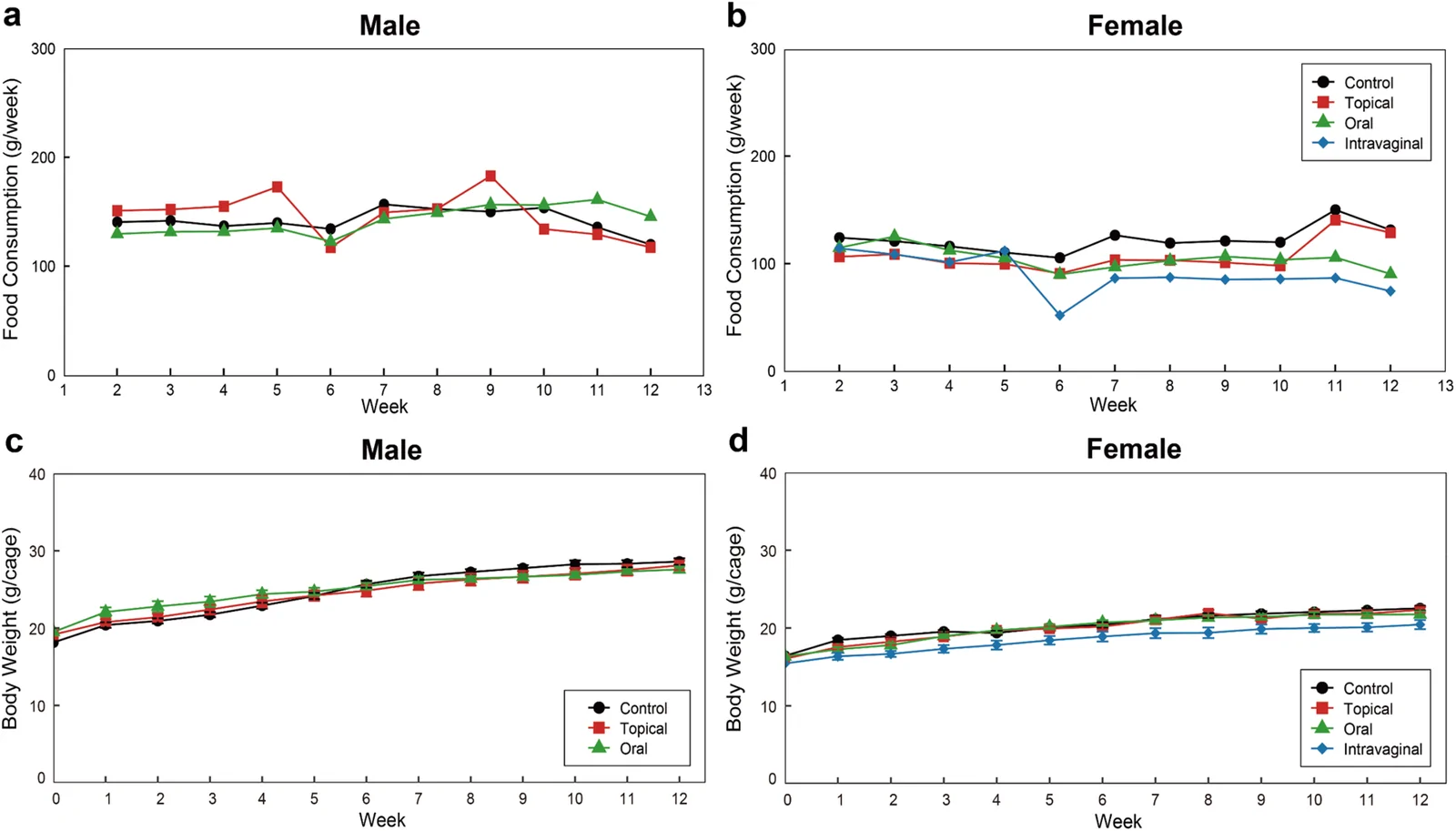
Food consumption and body weight during Nowarta110 treatments. Weekly food consumption per cage for (a) male and (b) female mice during different treatments. Average body weight for (c) male and (d) female mice during different treatments.

Across the study period, food consumption per cage (five animals per cage) in both male and female mice remained within normal ranges (mean weekly intake of 141.1 ± 15.0 g per cage for males and 106.9 ± 17.7 g per cage for females; Fig. 4a and b), consistent with published reference values.^44,45^ Although some variability was observed over time, no treatment group exhibited a sustained reduction in food intake or a consistent treatment-related trend. Overall, food intake remained comparable between Nowarta110-treated animals and controls, suggesting no adverse effects on appetite or general well-being.

Consistently, body weight increased steadily over time in all groups and followed patterns comparable to those of their respective controls (Fig. 4c and d). No treatment group exhibited sustained weight loss or suppression of expected weight gain. Collectively, these findings indicate that Nowarta110 treatments did not adversely affect normal growth during the study period.

### 3.6. Organ weight analysis

Organ weights measurements were evaluated as complementary endpoints to the hematological, serum biochemical, and urinalysis assessments, providing quantitative anatomical evidence that may reveal potential target-organ effects not detectable through circulating or urinary biomarkers alone. Alterations in organ weights can reflect early or subclinical toxicological responses, adaptive changes, or disrupted organ homeostasis before measurable functional impairment. Hence, when combined with previous analyses and histopathological findings, organ weight data can aid in identifying putative target organs and interpreting overall treatment tolerability.

**Fig. 5:**
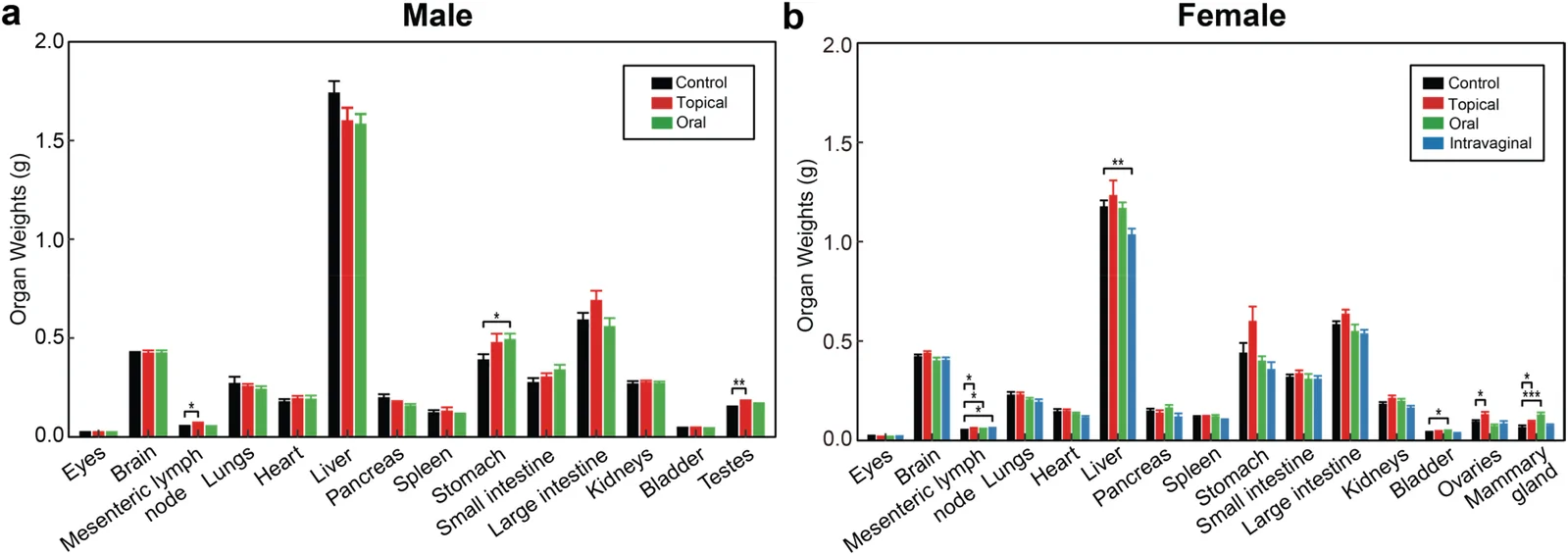
Organ weight analysis after Nowarta110 treatments. Organ weight measurements for (a) male and (b) female mice after different administration route treatments. (*), (**), and (***) indicate statistically significance with adjusted *p* < 0.05, *p* < 0.01, and *p* < 0.001, respectively.

As shown in Fig. 5, organ weights were broadly comparable between Nowarta110-treated groups and control animals in both male and female mice. Although statistically significant differences were observed in a limited number of organs, these findings were sporadic and did not follow a consistent pattern with respect to sex or route of administration. Notably, the majority of organs, including the eyes, brain, lungs, heart, pancreas, spleen, gastrointestinal tissues, and kidneys, showed no meaningful differences across treatment groups. Organs in which differences were noted (*e.g.* lymph nodes, liver, bladder, ovaries and mammary glands) did not exhibit clear or consistent trends. Overall, organ weights were largely comparable, with only a small number of isolated variations observed. Given the absence of consistency or dose- or route-related patterns, these changes were not considered treatment-related, indicating that Nowarta110 did not induce organ-level adverse effects under the conditions of this study.

### 3.7. Pathological findings

Histopathological evaluation was performed by licensed veterinarians to directly assess tissue-level evidence of potential treatment-related effects. In contrast to previous analyses, histopathology provides a definitive evaluation of cellular and structural integrity, enabling confirmation or exclusion of treatment-related tissue injuries and, when interpreted in combination with hematological, biochemical, urinalysis, and organ weight data, supporting the identification of target organs and the overall assessment of treatment tolerability.

**Fig. 6:**
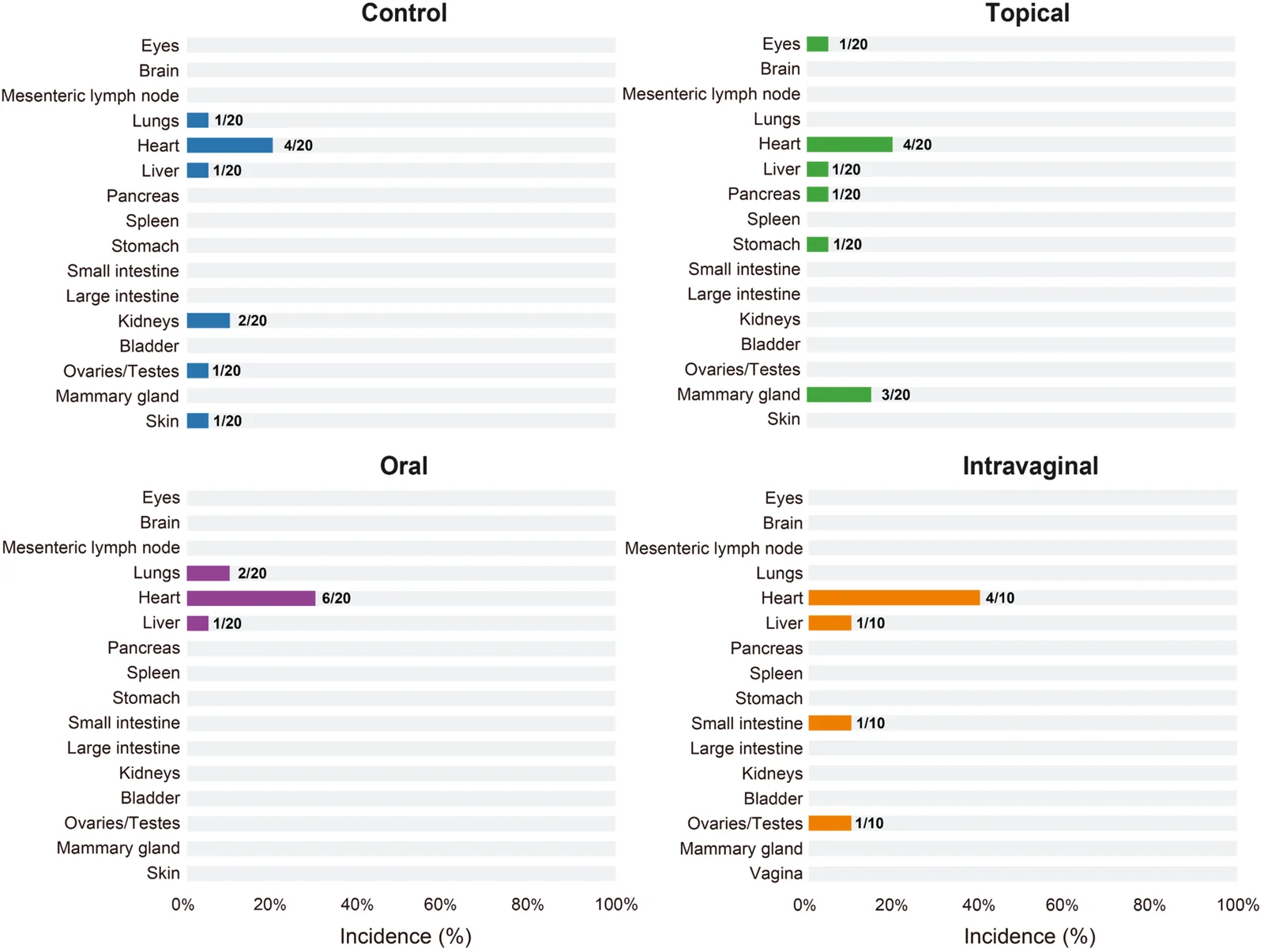
Histopathological findings after Nowarta110 treatments. Incidence of pathological findings across organs is shown for control, topical, oral, and intravaginal groups, expressed as the number of affected animals over the total examined.

Notably, histopathological examination revealed no findings attributable to repeated administration of Nowarta110 by topical, oral mucosal, or intravaginal routes (Fig. 6). No treatment-related lesions or neoplastic changes were identified. All observed findings were considered incidental in nature and occurred at comparable frequencies in control and treated animals, indicating a lack of treatment association. This was particularly evident for cardiac findings, which were detected across all groups, including controls. Importantly, Balb/c mice are known to exhibit a relatively high background incidence of spontaneous cardiac lesions, reported in approximately 10–60% of untreated animals,^46^ consistent with the incidence observed in the present study.

### 3.8. Plasma silver levels

A key safety concern associated with inorganic nanoparticles is their potential accumulation in highly perfused and fenestrated organs, which may lead to acute or chronic toxicities.^47,48^ This risk is generally reduced when nanoparticles are administered *via* non-intravenous routes, as was the case for the three administration routes evaluated in this study. Nevertheless, we investigated whether AgNPs could enter the systemic circulation following administration, as this leakage could contribute to systemic exposure and long-term particle retention.

Plasma samples were collected from all groups at multiple time points, ranging from 1 hour to 12 weeks after dosing, and silver concentrations were quantified by mass spectrometry. Notably, across all analyzed samples, silver levels were below the lower limit of quantification (22.6 ng mL^−1^), indicating that Nowarta110 did not reach the systemic circulation at measurable levels (Table S4). These findings further support the safety profile of the nanomedicine, demonstrating that non-intravenous administration does not result in systemic exposure.

## 4. Discussion

In the present study, the safety profile of the clinical-stage silver nanomedicine Nowarta110 was systematically evaluated following repeated dermal topical, oral mucosal, and intravaginal administration in mice. Hematology, serum biochemistry, urinalysis examinations, and animal well-being parameters, including body weight and food consumption, were monitored throughout the treatment period, while organ weights and histopathological examination were assessed as study endpoints. Across all evaluated parameters, no treatment-related adverse effects were identified.

This finding is particularly relevant given previous reports describing organ-specific toxicity of AgNPs, most notably affecting the liver, kidneys, and immune system, following intravenous administration and systemic exposure. In contrast to those reports, the present study revealed no consistent or treatment-related alterations in hematological parameters, serum biochemical markers, urinalysis findings, organ weights, and histopathological screenings after repeated local administration of Nowarta110. Although sporadic statistically significant differences were observed in selected circulating markers, these changes lacked consistency across sex, dose, route of administration, or exposure duration and were also observed in control animals. As such, they were considered to reflect normal biological variability rather than toxicologically meaningful effects. Histopathological evaluation further supported this conclusion, as no treatment-associated lesions, neoplastic changes, or organ-specific pathological patterns were identified. The incidental findings observed were comparable in nature and frequency to those typically reported in laboratory mice of similar age and strain.

To properly contextualize these findings, it is worth acknowledging several practical limitations in the current study. Although no consistent treatment-related changes suggestive of overt systemic immunotoxicity were identified across the evaluated hematological, organ weight, and histopathological endpoints, the present study was not designed as a dedicated immunotoxicity investigation and did not directly assess local immune responses or subtle functional immune changes. Similarly, the present study was designed as a repeated-dose safety assessment and therefore did not directly investigate the mechanisms underlying the biological interactions of Nowarta110. Rather, it evaluated the presence or absence of treatment-related macroscopic effects. Cellular uptake, local tissue interactions, biodistribution, and clearance pathways were not specifically assessed and should be investigated in dedicated mechanistic studies.

The physicochemical stability and transformation of Nowarta110 in circulation, including potential changes in the silver oxidation state, were not directly assessed, because silver concentrations in plasma remained below the limit of quantification by mass spectrometry following local administration. Consequently, insufficient silver-containing material was available for reliable recovery and post-exposure physicochemical or speciation analysis. The absence of quantifiable silver in plasma indicated that systemic exposure, if present, was below the analytical limit of quantification; however, it did not exclude local tissue deposition or persistence. Because tissue silver concentrations were not directly measured, local retention at the administration sites, low-level distribution to distant organs, and longer-term accumulation cannot be excluded.

The BALB/c mouse model enabled a controlled *in vivo* evaluation of repeated dermal, oral mucosal, and intravaginal administration of Nowarta110. These routes were selected to reflect its anticipated local use in humans. Nevertheless, species differences in anatomy, local physiology, immune responses, and nanoparticle disposition should be taken into account when considering the translational relevance of the findings. Potential reproductive and developmental toxicity following intravaginal administration was not evaluated and should be addressed in dedicated studies. The 12-week study period did not include an extended post-treatment follow-up and therefore does not allow conclusions regarding chronic toxicity beyond the study duration.

Nevertheless, within the scope of the current experimental design and the 12-week study period, the convergence of circulating markers, animal well-being parameters, histopathology, and toxicokinetic data consistently supports the absence of treatment-related toxicity under the tested conditions.

## 5. Conclusions

This study provides a comprehensive, multi-endpoint preclinical safety assessment of the silver nanomedicine Nowarta110 in mice. Under the conditions evaluated, repeated topical dermal, oral mucosal, and intravaginal administration over 12 weeks was well tolerated, with no treatment-related adverse effects identified across clinical observations, hematology, serum biochemistry, urinalysis, organ weights, histopathology, or toxicokinetic endpoints. No sex-related differences in response were detected, with male and female mice showing comparable findings across the assessed parameters. Plasma silver concentrations remained below the analytical limit of quantification at all evaluated sampling time points, indicating that systemic exposure, if present, was below quantifiable levels. Collectively, these findings support a favorable preclinical safety profile for Nowarta110 when administered locally by the routes investigated and provide a basis for its continued clinical development.

## Author contributions

BY (investigation, formal analysis, writing – original draft); RC (formal analysis); MW (formal analysis); LZ (formal analysis); OC (methodology); RZ (investigation); EMB (methodology); SR (methodology); BB (methodology); JK (methodology); JM (supervision); FK (supervision); TL (supervision); BH (supervision); BXH (conceptualization, supervision); RMP (conceptualization, supervision, writing – review & editing). All the authors read and approved the submitted version of the manuscript.

## Conflicts of interest

The authors have no relevant affiliations or financial involvement with any organization or entity with a financial interest in or financial conflict with the subject matter or materials discussed in the manuscript.

## Supplementary Material

NR-018-D6NR00957C-s001

## Data Availability

The data supporting this article have been included as part of the supplementary information (SI). Supplementary information: high-resolution electron microscopy; hematological and biochemical results; urinalysis and toxicokinetics after Nowarta110 treatments (PDF). See DOI: https://doi.org/10.1039/d6nr00957c.
