# Supplementary material for: Route- and sex-dependent toxicity of clinical-stage silver nanoparticles in mice
Source: Nanoscale. 2026 Sep 7;18(38):20684–94. doi: 10.1039/d6nr00957c (PMC13548031; doi:10.1039/d6nr00957c)
Supplement: Supplementary file 1 [file NR-018-D6NR00957C-s001.pdf]

Supplementary Information (SI) for Nanoscale.
This journal is © The Royal Society of Chemistry 2026

- SUPPORTING INFORMATION -

Route- and Sex-Dependent Toxicity of Clinical-Stage Silver Nanoparticles in Mice

Buerlan Yeerkenbieke,[1] Osmane Camara,[2] Ruxandra-Ioana Cipu,[1,3] Miryam Warwar,[1,3] Laura Zediu,[1,3] Rui Zhang,[1] Eva Miriam Buhl,[4] Stephan Rütten,[4] Bea Becker,[5] Jens Köhler,[5] Joachim Mayer,[3] Fabian Kiessling,[1] Twan Lammers,[1] Bo Han,[6] Ba Xuan Hoang [6,*] and Roger M. Pallares [1,*]

[1] Institute for Experimental Molecular Imaging, RWTH Aachen University Hospital, Aachen 52074, Germany

[2] Ernst Ruska-Centre for Microscopy and Spectroscopy with Electrons, Forschungszentrum Jülich GmbH, 52425 Jülich, Germany

[3] Faculty of Medical Engineering, National University of Science and Technology Politechnica Bucharest, Bucharest 011061, Romania

[4] Electron Microscope Facility, Institute for Pathology, RWTH Aachen University Hospital, Aachen 52074, Germany

[5] DWI – Leibniz Institute for Interactive Materials, Aachen 52074, Germany

[6] Department of Surgery, Keck School of Medicine, University of Southern California, Los Angeles, California 90033, United States

[*] Corresponding author: rmoltopallar@ukaachen.de , baxuanho@usc.edu

Table of contents

Figure S1. High-resolution TEM characterization of Nowarta110.........S2

Table S1. Hematological results in male and female mice at different time points.........S3

Table S2. Serum biochemical parameters in male and female mice at different time points.........S6

Table S3. Urinalysis results in male and female mice at different time points.........S9

Table S4. Silver plasma levels measured at multiple time points after different routes of administration.........S10

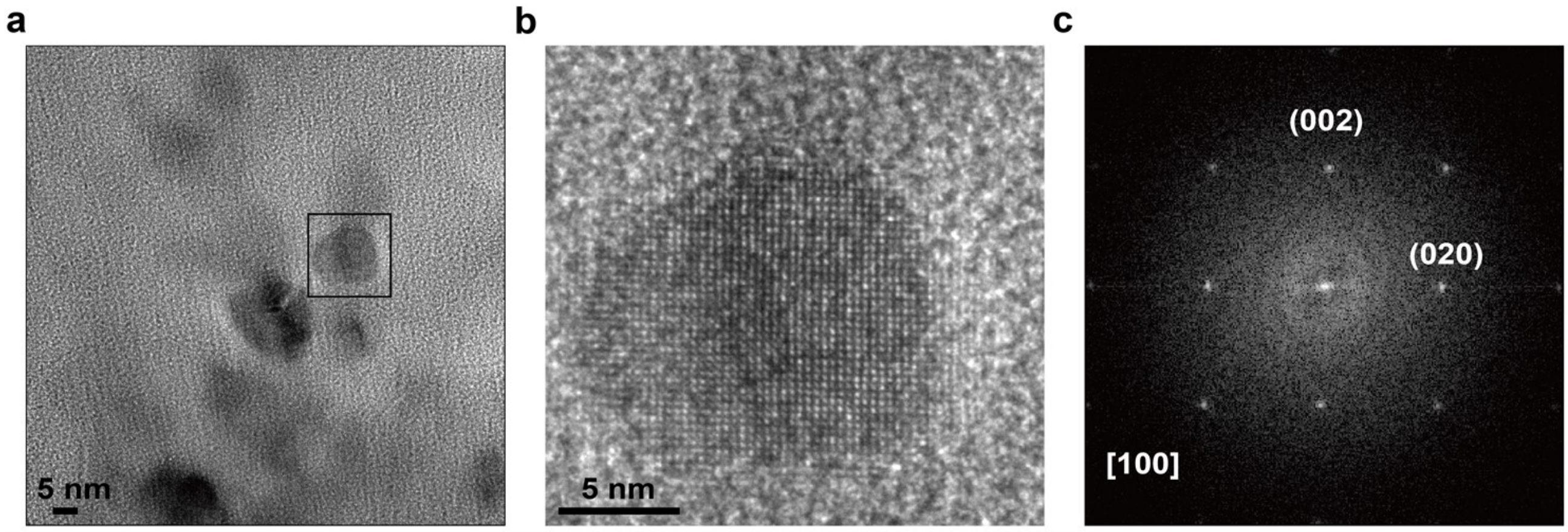

Figure S1. High-resolution TEM characterization of Nowarta110. (**a**) A representative high-resolution image of the nanoparticles. (**b**) An enlarged image of the selected region. (**c**) Corresponding fast Fourier transform patterns.

Table S1. Hematological results in male and female mice across control and treatment groups at different time points. Hematological indices are organized by sex, administration route, and evaluation time point (0, 5, and 12 weeks). Data are reported as mean ± standard deviation for each group (Control, Topical, Oral, and Intravaginal). Abbreviations: white blood cell count (WBC), lymphocytes (LYM), monocytes (MON), granulocytes (GRA), lymphocyte percentage (LY%), monocyte percentage (MO%), red blood cell count (RBC), hemoglobin (HGB), hematocrit (HCT), mean corpuscular volume (MCV), mean corpuscular hemoglobin (MCH), mean corpuscular hemoglobin concentration (MCHC), platelet count (PLT), and mean platelet volume (MPV). NA indicates not applicable.

Sex	Time	Metric	Unit	Control	Topical	Oral	Intravaginal
MALE	0 Week	WBC	$10^3/\mu L$	5.7 ± 2.2	4.4 ± 2.2	4.3 ± 3.8	NA
MALE	0 Week	LYMPH	$10^3/\mu L$	4.7 ± 1.9	3.6 ± 1.8	4.1 ± 3.1	NA
MALE	0 Week	MONO	$10^3/\mu L$	0.3 ± 0.2	0.2 ± 0.1	0.2 ± 0.2	NA
MALE	0 Week	GRAN	$10^3/\mu L$	0.8 ± 0.3	0.7 ± 0.4	0.5 ± 0.4	NA
MALE	0 Week	% LYMPH	%	80.5 ± 3.6	80.5 ± 5.6	83.6 ± 9.8	NA
MALE	0 Week	% MONO	%	4.3 ± 1.9	4.1 ± 2.4	3.0 ± 2.5	NA
MALE	0 Week	RBC	$10^6/\mu L$	8.2 ± 0.1	8.6 ± 0.3	9.0 ± 2.0	NA
MALE	0 Week	Hb	g/dL	15.1 ± 0.3	15.4 ± 0.3	14.7 ± 3.0	NA
MALE	0 Week	HCT	%	33.7 ± 0.7	34.6 ± 0.6	36.4 ± 8.1	NA
MALE	0 Week	MCV	fL	41.1 ± 0.7	40.2 ± 1.0	40.5 ± 1.4	NA
MALE	0 Week	MCH	pg	18.3 ± 0.3	17.9 ± 0.7	16.6 ± 2.6	NA
MALE	0 Week	MCHC	g/dL	44.8 ± 0.9	44.6 ± 1.4	40.9 ± 6.2	NA
MALE	0 Week	PLT	$10^3/\mu L$	397.0 ± 151.0	509.3 ± 211.3	557.3 ± 225.5	NA
MALE	0 Week	MPV	fL	5.8 ± 0.4	5.7 ± 0.3	6.1 ± 0.5	NA
MALE	5 weeks	WBC	$10^3/\mu L$	5.3 ± 1.7	4.0 ± 2.1	5.0 ± 2.1	NA
MALE	5 weeks	LYMPH	$10^3/\mu L$	3.1 ± 1.6	3.1 ± 1.3	4.4 ± 1.9	NA
MALE	5 weeks	MONO	$10^3/\mu L$	0.4 ± 0.3	0.2 ± 0.1	0.2 ± 0.1	NA
MALE	5 weeks	GRAN	$10^3/\mu L$	1.8 ± 1.3	0.8 ± 1.0	0.5 ± 0.4	NA
MALE	5 weeks	% LYMPH	%	61.0 ± 21.3	80.7 ± 12.9	87.4 ± 7.3	NA
MALE	5 weeks	% MONO	%	6.9 ± 3.9	3.8 ± 2.3	3.1 ± 2.0	NA
MALE	5 weeks	RBC	$10^6/\mu L$	8.5 ± 0.4	8.8 ± 0.3	8.4 ± 0.4	NA
MALE	5 weeks	Hb	g/dL	15.7 ± 0.8	16.0 ± 0.6	15.8 ± 0.7	NA
MALE	5 weeks	HCT	%	34.1 ± 1.7	34.5 ± 3.1	33.6 ± 1.1	NA
MALE	5 weeks	MCV	fL	40.0 ± 0.0	40.4 ± 0.8	40.0 ± 0.9	NA
MALE	5 weeks	MCH	pg	18.3 ± 0.3	18.2 ± 0.2	18.8 ± 0.3	NA
MALE	5 weeks	MCHC	g/dL	45.9 ± 0.7	45.0 ± 1.0	46.9 ± 1.4	NA
MALE	5 weeks	PLT	$10^3/\mu L$	531.0 ± 160.0	510.5 ± 165.4	351.5 ± 158.2	NA
MALE	5 weeks	MPV	fL	5.7 ± 0.3	5.9 ± 0.3	5.9 ± 0.6	NA
MALE	12w	WBC	$10^3/\mu L$	9.0 ± 4.4	6.8 ± 3.9	6.7 ± 5.0	NA

MALE	12 weeks	LYMPH	$10^3/\mu L$	7.1 ± 4.1	5.3 ± 3.5	5.4 ± 4.3	NA
MALE	12 weeks	MONO	$10^3/\mu L$	0.4 ± 0.3	0.4 ± 0.3	0.2 ± 0.1	NA
MALE	12 weeks	GRAN	$10^3/\mu L$	1.5 ± 0.7	1.0 ± 1.0	1.1 ± 0.9	NA
MALE	12 weeks	% LYMPH	%	75.0 ± 15.6	78.7 ± 15.3	73.8 ± 16.1	NA
MALE	12 weeks	% MONO	%	4.9 ± 3.4	5.2 ± 3.5	3.7 ± 2.5	NA
MALE	12 weeks	RBC	$10^6/\mu L$	8.0 ± 0.6	7.5 ± 1.2	8.2 ± 0.6	NA
MALE	12 weeks	Hb	g/dL	14.2 ± 1.1	13.3 ± 2.8	14.3 ± 1.1	NA
MALE	12 weeks	HCT	%	32.1 ± 2.6	30.2 ± 4.6	33.2 ± 1.5	NA
MALE	12 weeks	MCV	fL	40.1 ± 0.3	40.6 ± 1.7	40.6 ± 1.4	NA
MALE	12 weeks	MCH	pg	17.8 ± 0.4	17.6 ± 1.7	17.4 ± 0.5	NA
MALE	12 weeks	MCHC	g/dL	44.2 ± 0.7	43.5 ± 3.9	43.0 ± 1.7	NA
MALE	12 weeks	PLT	$10^3/\mu L$	276.1 ± 195.8	241.8 ± 129.2	346.1 ± 115.1	NA
MALE	12 weeks	MPV	fL	6.8 ± 1.0	6.7 ± 1.0	6.5 ± 0.5	NA
FEMALE	0 Week	WBC	$10^3/\mu L$	4.4 ± 2.5	3.0 ± 1.6	1.4 ± 0.8	5.7 ± 3.2
FEMALE	0 Week	LYMPH	$10^3/\mu L$	3.9 ± 2.3	2.5 ± 1.4	1.3 ± 0.9	4.7 ± 2.7
FEMALE	0 Week	MONO	$10^3/\mu L$	0.1 ± 0.1	0.1 ± 0.1	0.1 ± 0.1	0.2 ± 0.1
FEMALE	0 Week	GRAN	$10^3/\mu L$	0.3 ± 0.3	0.4 ± 0.2	0.3 ± 0.3	0.8 ± 0.4
FEMALE	0 Week	% LYMPH	%	86.6 ± 8.6	81.8 ± 5.9	73.1 ± 16.7	81.9 ± 5.4
FEMALE	0 Week	% MONO	%	2.8 ± 2.8	4.5 ± 3.2	3.0 ± 3.0	3.9 ± 1.7
FEMALE	0 Week	RBC	$10^6/\mu L$	7.5 ± 2.2	8.5 ± 0.4	8.3 ± 0.8	8.6 ± 0.1
FEMALE	0 Week	Hb	g/dL	13.4 ± 4.2	15.2 ± 0.5	15.3 ± 1.7	15.7 ± 0.43
FEMALE	0 Week	HCT	%	30.4 ± 9.0	34.0 ± 1.2	33.6 ± 3.1	34.2 ± 0.6
FEMALE	0 Week	MCV	fL	40.6 ± 0.5	39.9 ± 1.4	40.3 ± 0.8	40.0 ± 0.7
FEMALE	0 Week	MCH	pg	17.6 ± 1.1	17.8 ± 0.5	18.4 ± 0.7	18.3 ± 0.5
FEMALE	0 Week	MCHC	g/dL	43.4 ± 2.8	44.6 ± 1.1	45.6 ± 1.6	45.9 ± 1.0
FEMALE	0 Week	PLT	$10^3/\mu L$	294.6 ± 168.1	538.6 ± 168.0	563.3 ± 216.8	476.7 ± 126.9
FEMALE	0 Week	MPV	fL	6.7 ± 2.0	6.2 ± 1.2	5.8 ± 0.4	6.2 ± 1.0
FEMALE	5 weeks	WBC	$10^3/\mu L$	2.8 ± 1.2	3.0 ± 1.1	5.2 ± 2.0	5.7 ± 2.6
FEMALE	5 weeks	LYMPH	$10^3/\mu L$	2.5 ± 1.1	2.7 ± 1.1	4.5 ± 1.7	5.1 ± 2.3
FEMALE	5 weeks	MONO	$10^3/\mu L$	0.1 ± 0.1	0.1 ± 0.1	0.1 ± 0.1	0.2 ± 0.2
FEMALE	5 weeks	GRAN	$10^3/\mu L$	0.2 ± 0.2	0.2 ± 0.1	0.6 ± 0.5	0.4 ± 0.3
FEMALE	5 weeks	% LYMPH	%	89.8 ± 5.5	89.3 ± 4.0	86.5 ± 7.3	89.5 ± 4.2
FEMALE	5 weeks	% MONO	%	2.2 ± 1.7	3.1 ± 2.2	2.2 ± 1.6	3.1 ± 2.0
FEMALE	5 weeks	RBC	$10^6/\mu L$	8.3 ± 0.8	8.5 ± 0.4	8.5 ± 0.2	8.1 ± 1.5
FEMALE	5 weeks	Hb	g/dL	14.9 ± 2.5	15.5 ± 0.5	16 ± 0.3	15.0 ± 2.9
FEMALE	5 weeks	HCT	%	33.7 ± 3.2	34.6 ± 1.2	34.6 ± 0.9	32.4 ± 5.9
FEMALE	5 weeks	MCV	fL	40.5 ± 0.7	40.7 ± 0.9	40.8 ± 0.6	40.0 ± 0.0

FEMALE	5 weeks	MCH	pg	24.1 ± 20.3	18.1 ± 0.3	18.9 ± 0.4	18.4 ± 0.5
FEMALE	5 weeks	MCHC	g/dL	43.9 ± 4.3	44.7 ± 0.6	46.2 ± 0.9	46.0 ± 1.2
FEMALE	5 weeks	PLT	10^3/μL	372.7 ± 221.4	491.9 ± 175.5	527.7 ± 132.7	372.2 ± 164.9
FEMALE	5 weeks	MPV	fL	5.6 ± 0.2	5.7 ± 0.3	5.7 ± 0.2	5.8 ± 0.3
FEMALE	12 weeks	WBC	10^3/μL	9.7 ± 4.5	9.3 ± 3.5	8.1 ± 4.4	5.9 ± 3.7
FEMALE	12 weeks	LYMPH	10^3/μL	8.5 ± 4.1	8.2 ± 3.2	6.8 ± 3.7	4.5 ± 3.1
FEMALE	12 weeks	MONO	10^3/μL	0.5 ± 0.2	0.5 ± 0.3	0.4 ± 0.3	0.4 ± 0.4
FEMALE	12 weeks	GRAN	10^3/μL	0.8 ± 0.5	0.7 ± 0.4	0.9 ± 0.7	1.0 ± 0.6
FEMALE	12 weeks	% LYMPH	%	84.1 ± 15.1	86.5 ± 8.1	84.5 ± 8.3	75.3 ± 11.9
FEMALE	12 weeks	% MONO	%	5.4 ± 3.0	5.4 ± 3.4	4.4 ± 3.2	5.7 ± 2.3
FEMALE	12 weeks	RBC	10^6/μL	8.1 ± 0.5	7.4 ± 1.1	7.6 ± 1.2	8.0 ± 0.6
FEMALE	12 weeks	Hb	g/dL	14.8 ± 0.8	13.5 ± 2.3	13.3 ± 2.3	14.2 ± 1.0
FEMALE	12 weeks	HCT	%	33.1 ± 1.7	30.2 ± 4.4	30.8 ± 5.0	31.8 ± 2.2
FEMALE	12 weeks	MCV	fL	40.5 ± 0.7	40.9 ± 0.9	40.3 ± 0.7	39.6 ± 0.7
FEMALE	12 weeks	MCH	pg	18.2 ± 0.4	18.1 ± 0.6	17.4 ± 0.6	17.6 ± 0.5
FEMALE	12 weeks	MCHC	g/dL	44.7 ± 0.6	44.4 ± 1.8	43.1 ± 1.2	44.6 ± 1.2
FEMALE	12 weeks	PLT	10^3/μL	341.7 ± 107.2	235.1 ± 94.6	208.5 ± 144.0	325.5 ± 244.7
FEMALE	12 weeks	MPV	fL	7.4 ± 0.9	7.0 ± 0.7	6.1 ± 2.3	6.4 ± 1.0

Table S2. Serum biochemical parameters in male and female mice across control and treatment groups at different time points. Serum biochemical measurements are summarized by sex, administration route, and sampling time (0, 5, and 12 weeks). Values are expressed as mean ± standard deviation (SD) for each experimental group (Control, Topical, Oral, and Intravaginal). Abbreviations: alanine aminotransferase (ALT), albumin (ALB), alkaline phosphatase (ALP), amylase (AMY), calcium (Ca), phosphorus (PHOS), creatinine (CRE), globulin (GLOB), glucose (GLUK), sodium (Na), total bilirubin (T-BIL), total protein (TP), blood urea nitrogen (BUN), and potassium (K). NA indicates not applicable.

Sex	Time	Metric	Unit	Control	Topical	Oral	Intravaginal
MALE	0 Week	ALB	g/dL	3.5 ± 0.2	3.4 ± 0.2	3.4 ± 0.2	NA
MALE	0 Week	ALP	U/L	157.1 ± 10.4	164.5 ± 14.0	143.9 ± 37.0	NA
MALE	0 Week	ALT	U/L	66.9 ± 31.5	67.1 ± 46.2	171.8 ± 199.3	NA
MALE	0 Week	AMY	U/L	742.8 ± 56.8	745.4 ± 91.1	750.3 ± 89.7	NA
MALE	0 Week	BUN	mg/dL	19.2 ± 2.3	14.8 ± 1.1	18.5 ± 2.4	NA
MALE	0 Week	Ca	mg/dL	11.0 ± 0.2	10.6 ± 0.3	10.5 ± 0.4	NA
MALE	0 Week	GLOB	g/dL	1.5 ± 0.1	1.6 ± 0.2	1.5 ± 0.1	NA
MALE	0 Week	GLU	mg/dL	193.5 ± 24.7	214.6 ± 30.1	172.5 ± 62.2	NA
MALE	0 Week	K	mmol/L	4.8 ± 0.3	4.4 ± 0.4	5.2 ± 0.4	NA
MALE	0 Week	Na	mmol/L	147.4 ± 1.3	147.2 ± 2.4	149.8 ± 1.8	NA
MALE	0 Week	Phos	mg/dL	9.9 ± 2.3	7.0 ± 0.6	8.8 ± 1.6	NA
MALE	0 Week	T6BIL	mg/dL	0.2 ± 0.0	0.2 ± 0.0	0.2 ± 0.1	NA
MALE	0 Week	TP	g/dL	5.0 ± 0.1	4.9 ± 0.1	9.1 ± 13.3	NA
MALE	0 Week	CRE	mg/dL	0.1 ± 0.1	0.2 ± 0.1	0.1 ± 0.1	NA
MALE	5 weeks	ALB	g/dL	3.0 ± 0.3	3.2 ± 0.2	3.3 ± 0.3	NA
MALE	5 weeks	ALP	U/L	104.1 ± 11.2	98.4 ± 15.5	96.8 ± 13.3	NA
MALE	5 weeks	ALT	U/L	68.8 ± 37.0	91 ± 68.9	115.1 ± 70.0	NA
MALE	5 weeks	AMY	U/L	831.1 ± 106.7	773.5 ± 83.9	806.7 ± 112.9	NA
MALE	5 weeks	BUN	mg/dL	22.2 ± 2.2	21.3 ± 3.3	21.4 ± 3.0	NA
MALE	5 weeks	Ca	mg/dL	9.9 ± 0.4	9.9 ± 0.2	10.0 ± 0.4	NA
MALE	5 weeks	GLOB	g/dL	1.78 ± 0.1	1.7 ± 0.2	1.7 ± 0.2	NA
MALE	5 weeks	GLU	mg/dL	184.4 ± 29.3	160.4 ± 26.0	186.3 ± 34.3	NA
MALE	5 weeks	K	mmol/L	5.3 ± 0.4	5.2 ± 0.5	5.3 ± 0.2	NA
MALE	5 weeks	Na	mmol/L	147.5 ± 2.1	147.8 ± 2.0	148.6 ± 2.5	NA
MALE	5 weeks	Phos	mg/dL	7.0 ± 0.7	6.8 ± 0.6	6.7 ± 0.9	NA
MALE	5 weeks	T6BIL	mg/dL	0.2 ± 0.0	0.2 ± 0.1	0.2 ± 0.0	NA
MALE	5 weeks	TP	g/dL	4.9 ± 0.2	4.9 ± 0.2	4.9 ± 0.2	NA
MALE	5 weeks	CRE	mg/dL	0.1 ± 0.1	0.2 ± 0.1	0.1 ± 0.0	NA
MALE	12 weeks	ALB	g/dL	2.9 ± 0.4	3.2 ± 0.2	3.2 ± 0.3	NA
MALE	12 weeks	ALP	U/L	58.6 ± 15.0	60.8 ± 17.1	73.6 ± 10.4	NA

MALE	12 weeks	ALT	U/L	99.0 ± 101.7	206.8 ± 427.3	54.6 ± 22.3	NA
MALE	12 weeks	AMY	U/L	740.6 ± 65.8	747.7 ± 68.9	753.7 ± 77.4	NA
MALE	12 weeks	BUN	mg/dL	20.2 ± 2.0	21.2 ± 2.3	23.4 ± 6.7	NA
MALE	12 weeks	Ca	mg/dL	10.6 ± 0.7	9.7 ± 0.5	9.4 ± 1.6	NA
MALE	12 weeks	GLOB	g/dL	1.9 ± 0.3	1.7 ± 0.1	1.7 ± 0.1	NA
MALE	12 weeks	GLU	mg/dL	202.8 ± 31.6	197.0 ± 11.7	216.4 ± 31.3	NA
MALE	12 weeks	K	mmol/L	5.6 ± 1.2	4.8 ± 0.6	5.0 ± 0.3	NA
MALE	12 weeks	Na	mmol/L	149.9 ± 3.6	146.4 ± 2.3	147.8 ± 1.8	NA
MALE	12 weeks	Phos	mg/dL	10.4 ± 3.2	7.8 ± 1.8	7.1 ± 1.4	NA
MALE	12 weeks	T6BIL	mg/dL	0.2 ± 0.0	0.2 ± 0.1	0.2 ± 0.0	NA
MALE	12 weeks	TP	g/dL	4.8 ± 0.2	4.8 ± 0.2	4.9 ± 0.3	NA
MALE	12 weeks	CRE	mg/dL	0.2 ± 0.1	0.1 ± 0.1	0.2 ± 0.1	NA
FEMALE	0 Week	ALB	g/dL	3.5 ± 0.1	3.6 ± 0.2	3.9 ± 0.2	3.6 ± 0.2
FEMALE	0 Week	ALP	U/L	135.5 ± 16.5	144.6 ± 12.1	169.1 ± 16.7	142.4 ± 12.2
FEMALE	0 Week	ALT	U/L	63.7 ± 38.4	90.2 ± 95.5	84.9 ± 71.7	56.0 ± 19.5
FEMALE	0 Week	AMY	U/L	645.3 ± 40.9	618.0 ± 63.3	766.4 ± 179.9	663.4 ± 27.0
FEMALE	0 Week	BUN	mg/dL	20.0 ± 3.0	17.6 ± 2.4	25.1 ± 6.4	20.6 ± 3.3
FEMALE	0 Week	Ca	mg/dL	10.7 ± 0.4	10.8 ± 0.2	9.9 ± 0.4	10.3 ± 0.5
FEMALE	0 Week	GLOB	g/dL	1.3 ± 0.2	1.3 ± 0.2	1.2 ± 0.2	1.2 ± 0.2
FEMALE	0 Week	GLU	mg/dL	179.2 ± 23.8	194.3 ± 31.1	103.3 ± 49.3	166.7 ± 31.0
FEMALE	0 Week	K	mmol/L	4.9 ± 0.5	4.5 ± 0.8	4.8 ± 0.4	5.2 ± 0.4
FEMALE	0 Week	Na	mmol/L	146.9 ± 2.4	147.6 ± 1.8	149.3 ± 1.6	148.4 ± 1.3
FEMALE	0 Week	Phos	mg/dL	9.6 ± 1.9	8.8 ± 2.5	7.6 ± 1.0	7.8 ± 1.2
FEMALE	0 Week	T6BIL	mg/dL	0.2 ± 0.0	0.3 ± 0.1	0.2 ± 0.0	0.2 ± 0.0
FEMALE	0 Week	TP	g/dL	4.8 ± 0.2	4.9 ± 0.2	5.0 ± 0.2	4.7 ± 0.1
FEMALE	0 Week	CRE	mg/dL	0.1 ± 0.1	0.1 ± 0.0	0.1 ± 0.1	0.1 ± 0.1
FEMALE	5 weeks	ALB	g/dL	3.5 ± 0.2	3.5 ± 0.2	3.7 ± 0.1	3.7 ± 0.2
FEMALE	5 weeks	ALP	U/L	102.5 ± 11.9	103.2 ± 12.7	103.6 ± 14.9	99.7 ± 10.9
FEMALE	5 weeks	ALT	U/L	79.6 ± 95.3	60.3 ± 47.4	93.9 ± 102.8	68.0 ± 50.0
FEMALE	5 weeks	AMY	U/L	672.0 ± 42.8	672.9 ± 71.7	726.4 ± 26.8	671.2 ± 41.2
FEMALE	5 weeks	BUN	mg/dL	22.5 ± 3.7	22.5 ± 3.6	18.6 ± 2.5	19.5 ± 4.5
FEMALE	5 weeks	Ca	mg/dL	9.9 ± 0.2	9.9 ± 0.2	10.1 ± 0.3	9.9 ± 0.3
FEMALE	5 weeks	GLOB	g/dL	1.2 ± 0.1	1.3 ± 0.1	1.2 ± 0.1	1.1 ± 0.1
FEMALE	5 weeks	GLU	mg/dL	151.3 ± 30.4	152.5 ± 32.4	148.8 ± 32.5	176.2 ± 33.0
FEMALE	5 weeks	K	mmol/L	5.0 ± 0.5	4.8 ± 0.3	4.8 ± 0.5	5.2 ± 0.4
FEMALE	5 weeks	Na	mmol/L	145.6 ± 1.0	146.9 ± 1.6	146.8 ± 1.0	147.1 ± 1.5
FEMALE	5 weeks	Phos	mg/dL	7.4 ± 1.3	6.8 ± 0.6	6.3 ± 0.5	6.1 ± 0.7

FEMALE	5 weeks	T6BIL	mg/dL	0.2 ± 0.1	0.2 ± 0.0	0.2 ± 0.0	0.2 ± 0.0
FEMALE	5 weeks	TP	g/dL	4.8 ± 0.2	4.8 ± 0.2	4.9 ± 0.1	4.8 ± 0.2
FEMALE	5 weeks	CRE	mg/dL	0.2 ± 0.1	0.1 ± 0.1	0.2 ± 0.1	0.1 ± 0.0
FEMALE	12 weeks	ALB	g/dL	3.4 ± 0.3	3.4 ± 0.4	3.6 ± 0.1	3.7 ± 0.3
FEMALE	12 weeks	ALP	U/L	67.7 ± 15.9	58.2 ± 15.1	47.1 ± 21.7	59.2 ± 18.2
FEMALE	12 weeks	ALT	U/L	59.5 ± 19.7	128.0 ± 132.6	152.4 ± 149.7	90.2 ± 63.1
FEMALE	12 weeks	AMY	U/L	682.2 ± 54.4	636.1 ± 93.4	644.9 ± 87.3	749.0 ± 138.9
FEMALE	12 weeks	BUN	mg/dL	20.1 ± 2.6	20.5 ± 3.4	18.5 ± 1.9	21.3 ± 3.5
FEMALE	12 weeks	Ca	mg/dL	10.3 ± 0.2	9.4 ± 1.3	9.8 ± 0.4	10.2 ± 0.4
FEMALE	12 weeks	GLOB	g/dL	1.3 ± 0.2	1.2 ± 0.1	1.2 ± 0.2	1.1 ± 0.1
FEMALE	12 weeks	GLU	mg/dL	200.3 ± 42.7	185.7 ± 35.8	228.7 ± 34.0	177.7 ± 26.2
FEMALE	12 weeks	K	mmol/L	4.9 ± 0.4	4.4 ± 0.6	5.0 ± 1.0	4.7 ± 0.4
FEMALE	12 weeks	Na	mmol/L	145.5 ± 2.1	133.6 ± 27.8	142.5 ± 6.9	143.2 ± 3.4
FEMALE	12 weeks	Phos	mg/dL	8.4 ± 2.2	7.0 ± 1.6	7.7 ± 2.5	6.9 ± 2.1
FEMALE	12 weeks	T6BIL	mg/dL	0.2 ± 0.0	0.2 ± 0.0	0.2 ± 0.0	0.2 ± 0.0
FEMALE	12 weeks	TP	g/dL	4.7 ± 0.3	4.5 ± 0.5	4.9 ± 0.2	4.8 ± 0.2
FEMALE	12 weeks	CRE	mg/dL	0.1 ± 0.1	0.1 ± 0.1	0.2 ± 0.1	0.2 ± 0.1

Table S3. Urinalysis results in male and female mice across control and treatment groups at different time points. As identical urinalysis outcomes were observed for all individual animals, the data are presented in a grouped format. Each group included 10 animals.

Group	Time point	Sex	Urine pH	Protein	Blood	Leukocytes	Nitrite	Glucose	Ketone bodies	Bilirubin	Urobilinogen (mg/dL)
Control	0 week	Male	5	Normal	Negative	Negative	Negative	Negative	Negative	Negative	≤ 0.2
Control	0 week	Female	5	Normal	Negative	Negative	Negative	Negative	Negative	Negative	≤ 0.2
Control	5 weeks	Male	5	Normal	Negative	Negative	Negative	Negative	Negative	Negative	≤ 0.2
Control	5 weeks	Female	5	Normal	Negative	Negative	Negative	Negative	Negative	Negative	≤ 0.2
Control	12 weeks	Male	5	Normal	Negative	Negative	Negative	Negative	Negative	Negative	≤ 0.2
Control	12 weeks	Female	5	Normal	Negative	Negative	Negative	Negative	Negative	Negative	≤ 0.2
Oral	0 week	Male	5	Normal	Negative	Negative	Negative	Negative	Negative	Negative	≤ 0.2
Oral	0 week	Female	5	Normal	Negative	Negative	Negative	Negative	Negative	Negative	≤ 0.2
Oral	5 weeks	Male	5	Normal	Negative	Negative	Negative	Negative	Negative	Negative	≤ 0.2
Oral	5 weeks	Female	5	Normal	Negative	Negative	Negative	Negative	Negative	Negative	≤ 0.2
Oral	12 weeks	Male	5	Normal	Negative	Negative	Negative	Negative	Negative	Negative	≤ 0.2
Oral	12 weeks	Female	5	Normal	Negative	Negative	Negative	Negative	Negative	Negative	≤ 0.2
Topical	0 week	Male	5	Normal	Negative	Negative	Negative	Negative	Negative	Negative	≤ 0.2
Topical	0 week	Female	5	Normal	Negative	Negative	Negative	Negative	Negative	Negative	≤ 0.2
Topical	5 weeks	Male	5	Normal	Negative	Negative	Negative	Negative	Negative	Negative	≤ 0.2
Topical	5 weeks	Female	5	Normal	Negative	Negative	Negative	Negative	Negative	Negative	≤ 0.2
Topical	12 weeks	Male	5	Normal	Negative	Negative	Negative	Negative	Negative	Negative	≤ 0.2
Topical	12 weeks	Female	5	Normal	Negative	Negative	Negative	Negative	Negative	Negative	≤ 0.2
Intravaginal	0 week	Female	5	Normal	Negative	Negative	Negative	Negative	Negative	Negative	≤ 0.2
Intravaginal	5 weeks	Female	5	Normal	Negative	Negative	Negative	Negative	Negative	Negative	≤ 0.2
Intravaginal	12 weeks	Female	5	Normal	Negative	Negative	Negative	Negative	Negative	Negative	≤ 0.2

Table S4. Silver plasma levels measured at multiple time points after different routes of administration. Results are summarized by route, time point, and sex. All measured concentrations were below the lower limit of quantification (<22.6 ng/mL, BLOQ).

Administration	Time point	Sex	Number of mice	Result
Control	0 hour	Male	3	<BLOQ
Control	0 hour	Female	3	<BLOQ
Oral	1hour	Male	3	<BLOQ
Oral	3 hours	Male	3	<BLOQ
Oral	6 hours	Male	3	<BLOQ
Oral	9 hours	Male	3	<BLOQ
Oral	12 hours	Male	3	<BLOQ
Oral	24 hours	Male	3	<BLOQ
Oral	5 weeks	Male	3	<BLOQ
Oral	12 weeks	Male	3	<BLOQ
Oral	1 hour	Female	3	<BLOQ
Oral	3 hours	Female	3	<BLOQ
Oral	6 hours	Female	3	<BLOQ
Oral	9 hours	Female	3	<BLOQ
Oral	12 hours	Female	3	<BLOQ
Oral	24 hours	Female	3	<BLOQ
Oral	5 weeks	Female	3	<BLOQ
Oral	12 weeks	Female	3	<BLOQ
Topical	1 hour	Male	3	<BLOQ
Topical	3 hours	Male	3	<BLOQ
Topical	6 hours	Male	3	<BLOQ
Topical	9 hours	Male	3	<BLOQ
Topical	12 hours	Male	3	<BLOQ
Topical	24 hours	Male	3	<BLOQ
Topical	5 weeks	Male	3	<BLOQ
Topical	12 weeks	Male	3	<BLOQ
Topical	1 hour	Female	3	<BLOQ
Topical	3 hours	Female	3	<BLOQ
Topical	6 hours	Female	3	<BLOQ
Topical	9 hours	Female	3	<BLOQ
Topical	12 hours	Female	3	<BLOQ
Topical	24 hours	Female	3	<BLOQ
Topical	5 weeks	Female	3	<BLOQ

Topical	12 weeks	Female	3	<BLOQ
Intravaginal	1 hour	Female	3	<BLOQ
Intravaginal	3 hours	Female	3	<BLOQ
Intravaginal	6 hours	Female	3	<BLOQ
Intravaginal	9 hours	Female	3	<BLOQ
Intravaginal	12 hours	Female	3	<BLOQ
Intravaginal	24 hours	Female	3	<BLOQ
Intravaginal	5 weeks	Female	3	<BLOQ
Intravaginal	12 weeks	Female	3	<BLOQ
